# An atlas of health inequalities and health disparities research: “How is this all getting done in silos, and why?”

**DOI:** 10.1016/j.socscimed.2020.113330

**Published:** 2020-11

**Authors:** Taya A. Collyer, Katherine E. Smith

**Affiliations:** a University of Edinburgh, School of Social and Political Science, 15a George Square, Edinburgh, EH8 9LD, United Kingdom; b Monash University, Peninsula Clinical School, 2 Hastings Rd, Frankston, Victoria, Australia; c University of Strathclyde, School of Social Work and Social Policy, Lord Hope Building 141 St James Road, Glasgow, G4 0LT, UK

**Keywords:** Health disparities, Health inequalities, Health equity, Sociology of science, Disciplines, Scientific paradigms, Bibliometric analysis, Interviews

## Abstract

Research on health inequalities and health disparities has grown exponentially since the 1960s, but this expansion has not been matched by an associated sense of progress. Criticisms include claims that too much research addresses well-trodden questions and that the field has failed to gain public and policy traction. Qualitative studies have found researchers partly attribute these challenges to fragmentation resulting from disciplinary and methodological differences. Yet, empirical investigation (‘research on research’) is limited. This study addresses this gap, employing mixed-methods to examine, at scale, how and why this field is defined by insular research clusters. First, bibliometric analysis identifies and visualizes the 250 most-connected authors. Next, an algorithm was used to identify clustering via citation links between authors. We used researcher profiling to ascertain authors' geographical and institutional locations and disciplinary training, examining how this mapped onto clusters. Finally, causes of siloing were investigated via semi-structured interviews with 45 researchers. The resulting ‘atlas’ of health inequalities and health disparities research identifies eight clusters of authors with varying degrees of connectedness. No single factor neatly describes observed fragmentation, health equity scholars exhibit a diverse disciplinary backgrounds, and geographical, institutional, and historical factors appear to intersect to explain siloed citation patterns. While the configuration of research activity within clusters potentially helps render questions scientifically manageable, it affirms perceptions of the field as fragmented. We draw on Thomas Kuhn and Sheila Jasanoff to position results within theoretical pictures of scientific progress. Newcomers to the field can use our findings to orient themselves within the many streams of health equity scholarship, and existing health equity scholars can use the atlas to move beyond existing geo-disciplinary networks. However, although stronger cross-cluster engagement would be likely to improve insights, the complex nexus of factors underlying the field's structure will likely make this challenging in practice.

## Highlights

• Our atlas of health equity research identified eight clusters with varying connectivity.
• No single factor neatly explained observed fragmentation.
• Authors' geographic location, discipline, institution seem to influence citation flows.
• Author-level bibliometric analysis helps interrogate the siloed structure of research.

## 1 Introduction

Health inequalities research and health disparities research (hereafter ‘health equity research’) aims to understand, explain and reduce the unequal distribution of health across groups defined by social demographic factors, such as education, income and ethnicity. Previous studies chart the exponential accumulation of articles on this topic from 1966 onwards, demonstrating wide geographical interest, though a dominance of US and UK contributions (Bouchard et al., 2015; Cash-Gibson et al., 2018). However, growth in research has not been matched by improvement in health equity. Many countries have charted persistent health gaps between their most and least marginalized groups (Pool et al., 2017; Smith et al., 2015). Although slightly more positive accounts have recently emerged from some Western European countries (Mackenbach et al., 2018), researchers have nonetheless been critical of the field's lack of success. While some of these critiques emphasize the failure of political and policy actors to respond to available evidence (McCartney et al., 2013; Qureshi, 2013), there have been at least three sets of charges levied at researchers.

The first relates to failure to adequately translate research into policy and practice (often directed jointly at researchers and policymakers). Here, for example, Lynch argued scholars and policymakers employ health inequalities as a medicalized frame for discussing social inequalities and, in so doing, made the issue politically more appealing but ‘technically quite difficult to solve’ (Lynch, 2017). Multiple authors highlight the lack of coalitions connecting health equity scholars to those with advocacy, media and policy expertise (Smith et al., 2015; Bambra et al., 2011). Other critiques focus on researchers' perceived failure to engage with the public, noting that perceived public preferences often inform what is deemed politically feasible (Garthwaite et al., 2016; Smith, 2013).

The second set of criticisms relate to research content. Here, charges include a tendency to investigate well-trodden questions (e.g. describing patterns and causal pathways), while neglecting the impacts of policies and interventions (Bambra et al., 2011; Smith, 2013; Garthwaite et al., 2016). For example, Bambra and colleagues criticize researchers and policymakers for failing to collaborate in ways that enable the effects of policy changes on health inequalities to be adequately assessed (Bambra et al., 2011). While a survey of health inequalities researchers working in the UK suggests researchers themselves feel the field has been preoccupied with downstream risk-factors, such as smoking, whilst producing insufficient research on upstream, structural and social determinants (Smith and Eltanani, 2014). This imbalance has, in turn, been linked to researchers’ perceptions of what is likely to be funded (Smith, 2010), suggesting research funders also shape the field.

The third set of issues relate to perceived lack of connectivity. Division is evident even in the characterization of fundamental terms and concepts. As Bouchard et al.'s (2015) bibliometric analysis demonstrates, the terms ‘health inequities’, ‘health inequalities’ and ‘health disparities’ are used inconsistently (sometimes interchangeably), suggesting the presence of distinct scientific - perhaps epistemic - communities. The boundaries separating these communities have, in turn, been linked to geographical norms (Graham, 2004), disciplinary preferences (Bouchard et al., 2015) and ‘issue framing’ (Gamble and Stone, 2006). The latter dimension appears particularly confused; while there is some consensus that the term ‘health disparities’ is descriptive, some claim ‘health inequalities’ better signals health differences as unfair and unjust (Gamble and Stone, 2006), while others claim that only ‘health inequities’ is imbued with this moral dimension (Ward et al., 2013; Braveman, 2006).

Beyond differences in terminology, qualitative research suggests disciplinary divisions are particularly important. Bartley's analysis of research on unemployment and health in the UK highlighted the importance of differences between economics, statistics and sociology, noting that these cut across the boundary between research and policy (Bartley, 1992). More recently, Wade and Stone's (2010) reflections on teaching health disparities note that sociology and economics tend to approach this issue with entirely different questions and assumptions. Garthwaite and colleagues' (2016) study exploring how researchers feel the field should progress identified three epistemological clusters, each supporting distinct ways forward.

These three sets of critique are interrelated, with the first two being at least partially connected to the third. For example, fragmentation arising from geographical and disciplinary differences has been connected to claims that the field lacks clear advocacy-coalitions (Smith, 2013). While methodological preferences of dominant disciplines (especially epidemiology), and the lack of interdisciplinary collaboration, have been charged with contributing to the field's ongoing production of ‘partial investigations’ (Garthwaite et al., 2016). In other words, a belief that health equity research is fragmented is viewed as problematic for the field in multiple ways.

Understanding how and why health equity research is siloed therefore seems important. Yet there has been little empirical examination of the field (sometimes referred to as ‘research on research’) and even less examining disciplinary diversity. This study helps address this gap, employing mixed methods to examine, at scale, how the field is organised within citation-space and to ascertain the roles of disciplinary, geographical and institutional factors in the establishment and maintenance of this observed structure. By employing a bibliometric approach, we provide a much broader view of divisions within the field than qualitative studies published to date. We also move beyond existing bibliometric analyses by analyzing the connectivity of authors, not papers. Our analysis provides fresh empirical support for claims that the field is fragmented and, for the first time, identifies notable clusters of authors within the field and their relationship to one another in citation-space. Researcher profiling and qualitative interviews with researchers featuring in the bibliometric analysis are then used to shed light on these findings, demonstrating that disciplines, institutions and geographies have intersected to contribute to the emergence of distinct clusters (or ‘silos’). These findings can help newcomers to the field orient themselves within the literature, and also have important implications for those seeking to promote interdisciplinary collaboration within health equity research.

## 2 Methods

To avoid reliance on low-quality institutional affiliation data (Rafols and Meyer, 2007) or a focus on highly-cited researchers, bibliometric analysis was employed to identify the 250 most-connected authors within health equity research. This number was felt to be visually manageable, while including a range of career stages and locations.

We selected Scopus, the largest academic database (Mongeon and Paul-Hus, 2016) as the source database and article titles, abstracts and keywords were searched using the search string below, to extract records dated between 1976 and 2016. No geographical or language restrictions were applied. However, the use of English search-terms restricted results to those with an English title, abstract and/or keywords.

((“health inequ*“) OR (“health equal*“) OR (“health equit*“) OR (“health disparit*“) OR (“social determin* of health”))

As bibliometric analysis at the author level requires a high standard of data-hygiene (Wagstaff and Culyer, 2012), records were read into an SQL database for cleaning (correcting misspellings, merging authors appearing under multiple names, and distinguishing authors sharing names).

The bibliometric analysis aimed to visualise health equity research and to uncover patterns in citation flows that enhance understanding of the extent to which members of the field are integrated or segregated within disciplinary, geographic or institutional silos. Authors with five or more publications meeting search criteria were eligible for inclusion, and distances between author pairs calculated via direct citation (how many times Author A cites Author B). This method was selected over analysis of co-authorship as, when contrasted with article authorship, citations more completely reflect the material on which scholars draw and the literatures to which they feel their work connects.

A clustering algorithm was then employed to highlight regions of the network where a high proportion of citations are local. Waltman & Van Eck's Smart Local Moving algorithm (2013), was used, which initialises by assigning each author to a cluster of which they are the only member (i.e. 250 clusters where n = 1), and moves authors between clusters until the proportion of citations within groupings is maximised. Therefore, an author may cite authors from any cluster, but they tend to be cited (and/or to cite) authors from their own cluster.

Next, we used what we term ‘researcher profiling’ to ascertain the institutional and geographical location of authors, as well as their disciplinary training. To gather this information we undertook online searches and, where necessary, contacted researchers directly via email. Since observed diversity in disciplinary training could arise due to variation in course titles between countries, institutions, or over time, we classified researchers' highest degree (e.g. PhD) into Subject Categories, using Porter and Rafols' (2009) mapping. Following Jost (2007), the Shannon Number Equivalent (SNE) index is presented as a measure of disciplinary diversity within clusters. This can be interpreted as the number of equally-represented disciplines required to achieve the observed diversity of each cluster. Bibliometric analyses were conducted using VOSviewer (Van Eck and Waltman, 2017) and diversity measures calculated using Stata (Statacorp).

**Fig. 1:**
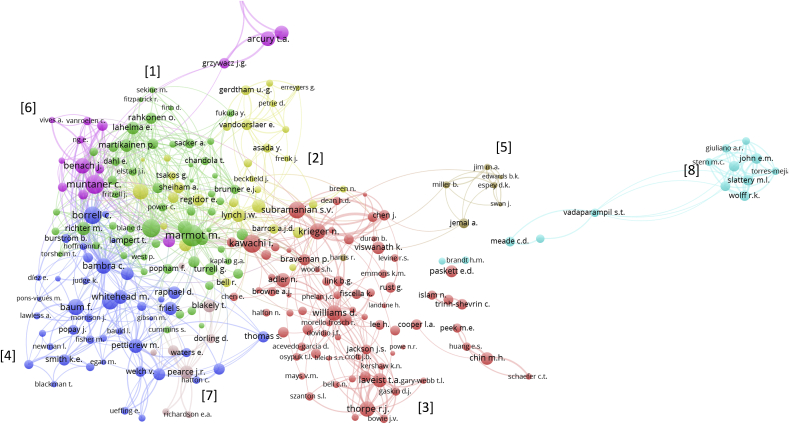
**The 250 most-connected health equity researchers.** Nodes represent authors who have published at least 5 papers with relevant keywords. The size of the node/circle represents the number of papers each author has published. Width of lines indicates the number of citations between authors. The colour of the nodes represent different clusters (numbered 1–8) of authors detected via algorithm.

In addition, the first author undertook semi-structured interviews with 45 researchers appearing in the network, to better understand their disciplinary backgrounds, career development and views about health inequalities/disparities research. Interviewees were shown an early version of Fig. 1, enabling them to reflect on clustering. Interviews were conducted using a themed interview schedule, via in-person meetings (n = 15), phone-calls (n = 3) and video-calls (n = 28). 113 researchers were invited to participate, and, with the exception of Cluster 5 (from which no interviews resulted), at least 3 representatives from all clusters were interviewed. Interviews were recorded and transcribed verbatim by the first author and thematically coded using the R Package RQDA (Huang, 2016) to aid analysis. This research project, including interviews, received ethical approval from the University of Edinburgh School of Social and Political Science.

This study does not present the entire, global health equity research network, but the 250 most densely-connected set of authors, meaning that satellite communities not citing any author in Fig. 1 are excluded. Our long time-scale may introduce bias toward late-career, established researchers. However, as over 80% of individual papers analysed were published after 2008, this impact seems likely to be minimal. Finally, as researchers’ PhD discipline may not accurately reflect the discipline of their current research output, some disciplinary communities may remain undetected.

## 3 Results

We begin by providing an overarching summary of the visual network produced via the bibliometric analysis, before exploring geographical and disciplinary dimensions of the network. Together with the researcher profiling, 45 interviews and our analysis of the cited papers, this informs the subsequent brief descriptive account of the eight clusters within the network. The final section of the results then draws directly on the interview data in attempt to understand this clustering in more depth.

### 3.1 Network overview

29,212 papers containing relevant keywords were extracted, representing over 8500 authors. Citation flows between pairs of authors were analysed, the 250 most-connected authors identified, and arranged such that authors with strong citation links are located close together, producing Fig. 1. Hence, Fig. 1 depicts the 250 most-connected authors publishing research in English about health equity between 1976 and 2016 (hereafter, “researchers” or “authors”). Despite spanning 40 years, 25,165 of papers (86%) were published after 2008, reflecting the exponential increase noted by existing reviews (Cash-Gibson et al., 2018; Bouchard et al., 2015).

**Fig. 2:**
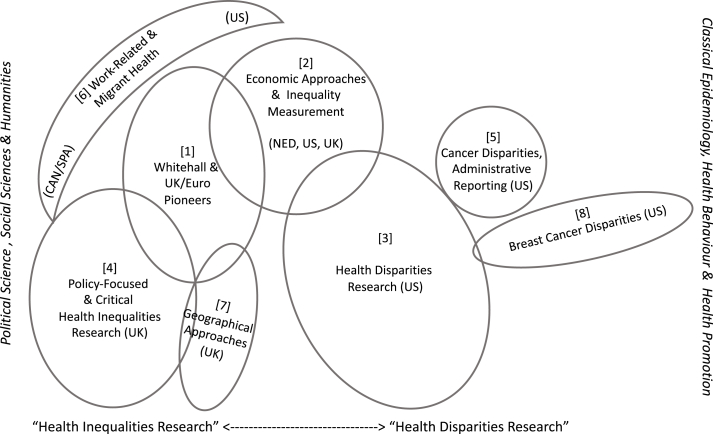
Eight clusters of health equity research.

Fig. 2 is a simplified version of Fig. 1, highlighting the eight clusters. The left and right halves of Fig. 1, Fig. 2 have different spatial arrangements and citation structures; the left is made up of five densely-connected, partly-overlapping clusters (labelled 1, 2, 6, 7 and 4 in Fig. 1, Fig. 2); while the right comprises three non-overlapping clusters with relatively sparse interconnectivity (labelled 5, 8 and 3). Generally (though with exceptions), researchers from the US appear on the right hand side of Fig. 1 and researchers from the UK, Europe, Australia, Canada and Europe appear on the left. In the following sections we investigate authors’ geographic distribution, and other author attributes which seem to overlay and intersect with geography to produce the structure of Fig. 1.

### 3.2 Network geography

The geographic location of network members is summarised in Table 1. Reflecting the findings of studies focused on the geographical spread of the field (Bouchard et al., 2015; Cash-Gibson et al., 2018), two-thirds of researchers are based in the US or UK, and an additional 13% in Canada and Australia. The remainder of the network comprises researchers from Europe, Scandinavia, Latin America and South-East Asia.

**Table 1:** Network members’ geographical location.

Country	Count	%
US	108	43%
UK	59	24%
Canada	19	8%
Australia	12	5%
Netherlands	8	3%
Germany	7	3%
Spain	7	3%
Sweden	5	2%
Brazil	3	1%
Finland	3	1%
Norway	3	1%
Belgium	2	1%
Chile	2	1%
Japan	2	1%
South Korea	2	1%
New Zealand	2	1%
Switzerland	2	1%
Other	4	2%
Grand Total	250	100%

### 3.3 Network disciplines

Table 2 provides a breakdown of first degree and highest degree by subject category, providing the first detailed picture of the disciplinary backgrounds underpinning the health equity research field. A wide range of disciplines are represented in network members' first degrees. Perhaps unsurprisingly, there is less variation in researchers' PhD or other highest degree (Juris Doctor, DPhil, etc), with the most common category being ‘Public, Environmental & occupational health’ (which includes epidemiology, health promotion, health behaviour and health education). This is followed by sociology, medicine, psychology, economics/health economics, and political science. Almost one in five network members have a medical qualification.

**Table 2:** First Degree and PhD/Highest Degree by Subject Category Data obtained from CVs, online profiles, and (where necessary) from researchers directly via email.

Subject Category	First Degree	PhD (Or Highest Degree)
Public, environmental & occupational health	6	74
Sociology	27	32
Medicine, general & internal	44	22
Psychology	26	13
Economics	11	12
Political Science	8	11
Geography	12	8
Social sciences, biomedical	3	8
Statistics & probability	3	8
Psychology, clinical		7
Nursing	8	5
Demography		4
Health policy & services		4
Biochemistry & molecular biology	2	3
Health care sciences & services	8	3
Medicine, research & experimental	2	3
Social sciences, interdisciplinary	7	3
Anthropology	2	2
Behavioral sciences		2
Dentistry, oral surgery & medicine	3	2
Ecology	1	2
History	7	2
Social work	2	2
Urban Studies		2
Biology	15	1
Business		1
Communication		1
Education & educational research		1
English/Literature	7	1
Family studies		1
Genetics & heredity	1	1
Information science & library science	1	1
Law		1
Nutrition & dietetics		1
Philosophy		1
Planning & development		1
Chemistry	6	
Design	1	
Engineering	2	
Management	1	
Mathematics	5	
Microbiology	2	
Multidisciplinary sciences	3	
Neurosciences	1	
Pharmacology & pharmacy	2	
Public administration	3	
Religious Studies	2	
Veterinary sciences	1	
Zoology	2	
Unknown	13	4
Total	250	250

Such varied undergraduate training suggests the field potentially incorporates diverse ideas about health, and health equity. However, the most common ideas are likely to be shaped by the dominant disciplines which are, in order, public health, sociology, medicine, psychology, economics, and political science.

In the following section we explore the clusters in Fig. 1, Fig. 2, including via examination of disciplinary diversity.

### 3.4 Network clusters

Fig. 1 contains the set of 250 most-connected health equity scholars, in terms of citation links. A large volume of citations flow across cluster boundaries, however the eight clusters visualised represent network regions where a high proportion of citations are local (i.e., within-cluster). Combining demographic data with the citation network allows investigation of the extent to which disciplinary diversity is uniform across Fig. 1, or maps onto the clusters (suggesting disciplinary silos). Fig. 2 is a simplified version of Fig. 1, highlighting the arrangement and general character of the eight clusters. The labels in Fig. 2 reflect analysis presented in this section, which considers what research profiling and interviews revealed about the intersecting influence of disciplines, geography, history, and research focus on cluster formation.

Table 3 contains key details regarding each cluster, including size, proportion in the US/UK, proportion with a medical qualification, and members’ highest degree by subject category. The US/UK breakdown is presented as while these countries account for over 50% of the network, they are not equally represented within any cluster, and some clusters are dominated by either the US or UK.

**Table 3:** Network Cluster characteristics.

Cluster	1	2	3	4	5	6	7	8	Total
(n)	57	31	76	42	7	15	8	14	250
Number Equivalent Shannon Index	9.68	4.53	9.68	13.07	3.6	8.33	4.01	3.42	14.15
% US	7%	19%	96%	0%	86%	26%	0%	93%	43%
% UK	40%	19%	1%	52%	0%	0%	88%	0%	24%
% Any Medical Degree	18%	32%	20%	12%	29%	40%	13%	7%	20%
Median year of authors' first included publication	1999	2002	2004	2005	2005	2006	2008.5	2011.5	2004
Earliest first included publication	1985	1997	1993	1983	2002	1999	2001	2003	1983
***Subject Category: PhD/Highest Degree***			**(% of cluster)**						**(% network)**
Public, Environmental & Occupational Health	19%	45%	36%	19%	43%	20%	0%	64%	30.00%
Medicine, general & internal	4%	6%	14%	5%	29%	13%	13%		8.80%
Medicine, research & experimental					14%	13%			1.20%
Nursing			5%					7%	2.00%
Dentistry, oral surgery & medicine		3%	1%						0.80%
Nutrition & dietetics			1%						0.40%
Psychology	11%		5%	5%		7%			5.20%
Psychology, clinical			8%	2%					2.80%
Behavioral sciences			3%						0.80%
Education & educational research				2%					0.40%
Statistics & probability	7%		1%		14%	7%		7%	3.20%
Biochemistry & molecular biology	4%							7%	1.20%
Ecology	2%						13%		0.80%
Biology								7%	0.40%
Genetics & heredity								7%	0.40%
Health policy & services			3%			13%			1.60%
Health care sciences & services	2%			5%					1.20%
Planning & development				2%					0.40%
Family studies						7%			0.40%
Demography	4%	3%		2%					1.60%
Urban studies	2%			2%					0.80%
Geography	2%		1%	5%			50%		3.20%
Communication			1%						0.40%
Business			1%						0.40%
Economics	2%	29%	3%						4.80%
								
Social sciences, biomedical	4%		1%	7%			13%		2.80%
Social sciences, interdisciplinary	2%			2%		7%			1.20%
Social work	2%		1%						0.80%
Sociology	26%	6%	9%	17%			13%		12.40%
Political science	4%	3%	3%	14%					4.40%
Anthropology						13%			0.80%
Law			1%						0.40%
History		3%		2%					0.80%
Literature				2%					0.40%
Philosophy				2%					0.40%
Information science & library science				2%					0.40%
Unknown	7%								2.00%
**Total**	**100%**	**100%**	**100%**	**100%**	**100%**	**100%**	**100%**	**100%**	**100.00%**

Table 3 also reports the SNE index, reflecting both the number of disciplines and evenness of their representation within clusters. The entire network of 250 authors has SNE of 14.15, equivalent to approximately 14 equally-represented disciplines (or, 250/14–18 examples of each discipline). Only Cluster 4 is as diverse as the wider network, suggesting a degree of disciplinary sorting or concentration. This possibility is explored below, as we examine each cluster in turn, in order of increasing median year of first publication.

*Cluster 1: Whitehall Investigators & Health Inequalities Pioneers (UK/Europe).*

The network's oldest cluster (by median entry to the field) is comprised largely of researchers from the UK and Europe who began studying health inequalities during the 1980s and 1990s. The relatively high diversity score reflects the wide-ranging backgrounds of these early inequality scholars, which included many social scientists. This cluster is especially notable for its high proportion of Sociology PhDs, making up just over a quarter of the cluster, while psychology, the social sciences and political science are also represented. The upper left part this of cluster includes several sociologists from Scandinavia and Germany. Researchers toward the bottom of this cluster (overlapping Cluster 7) share a focus on place and health.

*Cluster 2: Economic approaches & Measurement of income inequity at scale (Netherlands, UK, US).*

Cluster 2 is comprised of two distinct regions, each with disciplinary features. Work in this cluster emerged in the early 2000s and addresses methodological issues arising from the international scaling-up of studies like those occurring in Cluster 1 (discussed in Section 3.4). Located at the top are economists from the Netherlands, Australia, Sweden, and the US, who have contributed advances in the measurement of health equity. Near the centre of the network are a group of epidemiologists and social epidemiologists, many of whom have medical or sociology backgrounds. Both regions share an interest in the *measurement* of health equity, and the ways in which money, income and prevailing economic conditions affect health, including dental and oral health. Cluster 2 is around three-quarters the size of Cluster 3, but demonstrates 35% of that cluster's diversity, reflecting the smaller number of disciplines represented.

*Cluster 3: Health Disparities Research (US).* Almost all (96%) of the network's largest cluster is located within the US, and half hold a PhD or other doctoral degree in Public health (including epidemiology) or medicine. Nursing, psychology and sociology PhDs are also represented, but political science and the humanities are absent. While the majority of authors in Cluster 3 have written about ethnic and racial disparities in health, researchers in the rightmost region of this cluster (near clusters 5 and 8) share a focus on racial and ethnic disparities in cancer outcomes. Researchers in the leftmost part of the cluster have a more mixed focus, including, for example, maternal and child health, drug use, mental health, and allergies. The top-left corner includes several researchers from the Harvard School of Public Health, and University of California San Francisco. These researchers have focused on the relationship between socioeconomic status and health, perhaps explaining their strong citation links with Clusters 1 and 2. The emergence of Cluster 3, and its links with Clusters 5 and 8, are discussed in Section 3.4.

*Cluster 4: Policy-Focused & Critical Health Inequalities Research (UK).*

Cluster 4 is unique within the network for its disciplinary diversity. The US is absent from this cluster, with 52% of members located within the UK, 19% in Australia and 17% in Canada. While the median entry to the field for authors was 2005, Cluster 4 generally developed alongside Cluster 1, throughout the 1980s and 1990s, as the strong citation links in Fig. 1 indicate. Members of this cluster have the network's most-diverse doctoral training, with an equivalent 13 equally-represented disciplines among just 42 members. The network's humanities PhDs and political science PhDs are concentrated here, and the social sciences are also well-represented. These trainings are consistent with the cluster's focus on macro or ‘upstream’ determinants, including political and corporate determinants. Many researchers in this cluster conduct qualitative research and have a theoretical emphasis in their work. The cluster covers topics such as health policy, lay knowledge and evidence synthesis. Together, these features reflect the conceptualisation of health within Cluster 4 as socially-situated. This cluster has strong citation links to Clusters 1, 7 and 6, but is sparsely linked with Cluster 3.

*Cluster 5: Racial and Ethnic Disparities in Cancer: Administrative Reporting (US).*

Cluster 5 is the network's smallest cluster and (like Cluster 8) is cancer-focused and located geographically within the US. Members of this cluster have co-authored highly-cited, national administrative cancer statistics reports which include cancer incidence and mortality for racial and ethnic subgroups (labelled with the keyword “health disparities” since 2002). Nearly one third of members have medical training, and remaining members have statistical, biomedical, or public health backgrounds, reflecting the output of this cluster. Most members are affiliated with either the National Cancer Institute, National Cancer Society, Centers for Disease Control, or National Institutes of Health (NIH).

*Cluster 6: Socio-Critical accounts of Work-Related and Migrant Health (US, Canada, Spain).*

Cluster 6 is a mix of researchers from Europe, Latin America and the US. Researchers in this cluster are linked via a joint focus on employment-related health disparities/inequalities, and migrant health. Several researchers are based in Barcelona or completed doctoral study in that city. An additional group are located at (or have passed through) the Wake Forest Department of Family Medicine, in the US. In terms of disciplinary training this is the most diverse of the small clusters (Clusters 5–8), containing a mix of researchers with medical, biomedical, family studies, health policy, psychology, and statistical backgrounds. The network's anthropology PhDs are also concentrated within this cluster, and this similar disciplinary profile perhaps explains the position in citation-space alongside Clusters 1 and 4, within which health is characterised as a socio-structural phenomenon.

*Cluster 7: Geographical Approaches: Inequalities in Place & Space (UK).*

This relatively new, chiefly UK-based cluster contains the majority of the network's geography PhDs. Located at the right-hand margin of Cluster 4, these researchers share a focus on spatial and geographic inequalities, environmental justice, and neighbourhoods. These eight researchers entered the field slightly later than researchers in other clusters, with a median first publication year of mid-2008.

*Cluster 8: Breast Cancer Disparities (US).*

This small cluster is comprised chiefly of contributors to a single project, the Breast Cancer Health Disparities study (Slattery et al., 2014), many of whom are (or were once) based at the University of Louisville, Kentucky, or University of Utah. This is the least-diverse cluster in terms of disciplinary background, with a concentration of researchers holding a PhD in Health Behaviour, Health Education or Health Promotion (consolidated within the Public Health Subject Category). Other members hold advanced degrees in statistics or biomedical science. This is the network's most recently established cluster, with members' median first publication in the field being mid-2011.

### 3.5 Exploring and explaining the eight research clusters

In this section, we draw on interview data to better understand how clusters have emerged and by what forces they are sustained in citation-space. As a reminder, we interviewed 45 network members and incorporated questions about an early version of Fig. 1. Although we use extracts illustrate specific points, we also draw on data collectively to draw out common explanatory narratives that appear to explain the evolution of the field and the clustering identified above.

#### 3.5.1 Landmark studies & advances in measurement

Major research projects have contributed to the form and disciplinary topology of Fig. 1. An early milestone of health inequalities research was the Whitehall cohort, established in 1967 and analysed from 1978 onwards to investigate the relationship between cardiovascular (and other) diseases and occupational social class within the British civil service (e.g. Rose and Marmot, 1981). The status of the Whitehall studies as paradigmatic examples of health equity scholarship is reflected in Fig. 1, as Whitehall investigators and collaborators make up much of Cluster 1, and occupy a central position in the network.

Dutch researcher Johan Mackenbach spearheaded efforts to replicate Whitehall in Europe, beginning with the Dutch Longitudinal Study on Socio-Economic Health Differences, containing an explicit reference to Whitehall in its abstract (Mackenbach et al., 1994). Authors of similar single-country studies make up the top (left) half of Cluster 1. These replications provided comparable cohorts in several high-income countries and, therefore, the opportunity for cross-country comparison. This work was initiated by a group of economists, visible in Cluster 2. Throughout the late 1990s and early 2000s, the scaling-up of the field from single cohort studies to global mega-comparisons introduced methodological challenges, and an accompanying need for “valid measures and methods” (Manor et al., 1997). In response, a literature specific to the measurement of health equity emerged, largely authored by the economists in Cluster 2 and others located at the intersection of Clusters 1, 2 and 3 (Kakwani et al., 1997; Kawachi and Kennedy, 1997).

In sum, in the UK and Europe, paradigmatic epidemiological studies aiming to investigate how social class (measured by employment status) impacts health played a key role in the development of Clusters 1 and 2. A shared focus on social inequalities in health outcomes facilitated links between these two clusters.

#### 3.5.2 ‘Inequalities’ & ‘disparities’

The lack of citation links between Cluster 3 and the European/UK clusters is a conspicuous feature of Fig. 1. US researchers in Fig. 1 seem to mostly cite other US researchers, whereas researchers in Australia, Europe and Canada seem more interconnected. This may be a reflection of the specialized streams of research apparent in Fig. 1, and specialized communities of (for example) geographers, economists, clinical epidemiologists or social-scientists, unaware of potentially relevant publications from other streams. Alternatively, these different terms may signify varied framings of health equity as biomedical or sociological phenomena. In interviews, most interviewees were unable to explain this feature of Fig. 1, and interviewees in both network hemispheres commonly indicated they had little sense of the other, underlining their separation:

I have no idea who anyone is! I've never heard of any of those people! […]. How is this all getting done in silos? And Why??

Social Epidemiologist, USA, Network Cluster 3.

It is a bit hard to make sense of. There are a few names I've never heard of. I have to say […] I just can't think who these people are.

Geographer, UK, Network Cluster 7.

Some interviewees suggested space between Cluster 3 (US dominated) and other clusters is due to a combination of terminological differences and contrasting research foci, with UK based researchers generally studying ‘*inequalities’* between social classes, while researchers in the US tend to study ‘*disparities’* between racial and ethnic groups (Kawachi et al., 2002). However, as we go on to elaborate, this was only a partial explanation.

The separation between ‘inequalities’ and ‘disparities’ scholars in Fig. 1 appears to reflect the distinct origins and independent development of two research traditions. Several inequalities scholars interviewed were keen to highlight the historical context of Cluster 1, in the wake of the Black Report (Black, Morris et al., 1980), the first systematic effort by any national government to understand and explain health inequalities between social classes (Smith, 2013). The 1980s, 1990s were periods of intense activity for British health inequalities scholars, as they attempted to address gaps in understanding identified by the Black Report, while documenting the health impact of policies put in place by the Thatcher-led Conservative government that had rejected the Report's conclusions (e.g. Whitehead, 1987). Many members of Cluster 1 and 4 pursued research on health inequalities throughout this period, despite limited funding, when the idea of health inequalities, even the term itself, was politically controversial:

[It] was called the “health variations research program.” We were told we couldn't use “inequalities” because Margaret Thatcher didn't like the term, so it was dumped and we were “variations”.

Cluster 1 Researcher, UK.

Interview data suggests this struggle contributed to a shared sense of identity among these scholars, now passed to some students and collaborators present in Clusters 1, 4 and 7. There was resistance toward adopting any term other than ‘inequalities’ among these UK interviewees, because the term had been fought for by researchers perceived as pioneers:

Interviewer: Would you ever want to apply the term “Health Disparities” in the UK?

I would rather we stuck with ‘health inequalities’. […] During the Thatcher time, you weren't allowed to talk about health ‘inequalities’, I don't like dodging away from it.

Cluster 1 Researcher, UK.

There was also a sense that some UK interviewees considered ‘real’ health inequalities research as being concerned exclusively with social determinants, and restricted to network Clusters 1, 4 and 7:

There is that community, and there are factions within that community […] But we would all be seen as ‘Health Inequalities’ […] Some [are] more on the periphery, like [Researcher from Cluster 7] for example because [s/he's] more geography. [S/he] is more on the periphery, but a part of the family. And then there are almost like ‘interlopers’ of the mainstream […] they probably think that they're health inequalities researchers, but [they don't belong to] this group of people who *are* health inequalities, who have carried that trajectory within them, and have been shaped [by], and learned from, those pioneers.

Cluster 4 Researcher, UK.

In contrast, in the US, the importance of social factors in determining health outcomes was catalysed by studies in the 1980s noting differences in medical practice across (apparently) similar patient populations (McPherson et al., 1982). Responding to this unexplained variation in medical care, the US government commissioned the “Health, United States, 1983” report, which described, for the first time, significant differences in “the burden of death and illness experienced by blacks and other minority Americans as compared with the nation's population as a whole” (p ix). The dominance of the term “health disparities” arose from this motivating drive to understand the ‘gaps’ in observed health outcomes and health care access between minority and majority ethnic populations (e.g. AMA CEDA, 1990).

This emphasis continued into the 21st century, with health disparities research in the US tending to focus on healthcare (*e.g.*, Fiscella et al., 2000; Nelson, 2002), while the European concern with inequalities in health relating to social class also persisted (Marmot et al., 2012). Several interviewees noted the longstanding divide between scholars studying health inequalities (in class) and health disparities (in race):

Interviewer: Race is clearly very important to work on health disparities in the USA, it seems to be less of a focus in the UK?

P: Yeah I've noticed that […] and I'm not sure why that is. [..] I haven't gone as deeply into it as I might, because it is not, to be quite blunt, it doesn't interest me that much, in the UK context.

Cluster 4 Researcher, UK.

Here [in the US] it is very much on race, we don't talk about class here. […] There is so much focus on race here. Some of it makes sense, and some of it is really misguided and misses the point.

Cluster 3 Researcher, US.

US-based interviewees across the network, but most especially in Clusters 3 and 6, expressed concern regarding the way race is conceptualized in research, particularly within ‘mainstream’ epidemiology. NIH requirements to report findings by racial and ethnic subgroupings were positioned as contributing to the uncritical treatment of race in quantitative analyses:

Important now is to talk about race and ethnicity, to talk about race being a sociological concept and not a biological reality. […] In the US, because many of us are getting federal funds, one has to design research that covers human variation, and that is designated as sex, race and now age. […] This notion that you have to design it into your study means then that you have to be able to assign a value […]. In assigning that value then you have pretty much said “this is an entity, these different racial categories really are entities.”

Cluster 6 Researcher, US.

In summary, in Fig. 1 we see the lasting impact of the way research about health equity was conceptualized and initiated on either side of the Atlantic. Insights from interview data reveal the ongoing importance of the way the two fields originated in the 1980s and developed over time, trajectories reflected in the structure of Fig. 1. Perhaps more importantly, it is clear that ‘inequalities’ and ‘disparities’ are, in practice, not interchangeable terms for the same phenomena. To use one term aligns a project with a particular tradition of research, a group of pioneering investigators, and a historical conceptualisation of equity. Overlying geographic variation was disciplinary variation in use of these terms, discussed in the next section.

#### 3.5.3 Disciplinary diversity

The distribution of doctoral trainings is not uniform across the network, mostly due to the mix of humanities, political-, life- and social-sciences on the network's left side (which, as Fig. 2 illustrates, tend to study ‘health inequalities’), and the dominance of medical, statistical, health promotion and epidemiological backgrounds on the right side (where ‘health disparities’ dominates as the preferred term). Researchers with economic training are similarly concentrated in Cluster 2, as are the majority of geographers (Cluster 7). The small, US dominated clusters (5 and 8) contain many more cancer epidemiologists and health promotion scholars than the wider network, and represent a small number of institutions. Clusters 7 and 8 have the most recent median first publication date, and appear to represent regional communities of disciplinary and topical specialists. Clusters 1 and 3 (foundational clusters within the UK and US, respectively) have the same SNE diversity, though Cluster 1 might be considered more diverse as it is smaller.

One notable anomaly is Cluster 6, which includes a mix of ‘health inequalities’ and ‘health disparities’ researchers, appears on the health inequalities ‘side’ of Fig. 1 but does not include any members from the UK. Many members of Cluster 6 have social science backgrounds, and interviewees from this cluster explicitly framed the drivers of health inequity as socially-situated, which may explain the location of this group in citation space alongside clusters with strong social science membership. Nevertheless, interviewees in Clusters 1, 4 and 7 were almost universally unaware of the work proceeding in Cluster 6.

#### 3.5.4 Disease focus

In addition to varied use of terminology, and different disciplinary profiles, the two network hemispheres differ in their disease foci, reflected in the algorithmic detection of two cancer-specific, US-dominated citation clusters (5 and 8) on the network's right side. This difference may be at least partially due to the data availability landscape within the US throughout the 1990s. In 2002, six members of Cluster 3 expressed their frustration that “few or no socioeconomic data exist in most US public health surveillance databases” (Krieger et al., 2002). In the context of this scarcity, the Surveillance, Epidemiology and End Results registry (SEER) held high-quality cancer incidence and outcome data alongside patient demographics dating back to 1973 (National Cancer Institute), representing a crucial data source for health disparities scholars. Between 2000 and 2010, several members of Clusters 3, 5 and 8 utilised SEER data to demonstrate racial disparities in cancer screening, incidence, and health outcomes (*e.g.*, Singh et al., 2004). Analysis of SEER data to investigate disparities in cancer-related outcomes was also utilised to demonstrate best-practice methodology (Harper et al., 2008). Cancer disparities research continues to be well-funded in the US, and is supported institutionally via Comprehensive Cancer Centers. Many interviewees in the US suggested that the security of cancer-specific funding powerfully shapes research about health disparities:

Interviewer: Many US researchers who have appeared in my bibliometric network study cancer. Why do you think that is?

Because it's sexy. And well-funded.

Cluster 3 Researcher, US.

For interviewees in Clusters 3 and 8, there was a sense that studying cancer is a financial necessity, and that funding streams shape research questions:

We're pushed and funded to examine disparities in single disease conditions. And I am very guilty of this […] There are common underpinnings across a number of health behaviours, health conditions, health outcomes […] but in the US it is very disease focused, that is how our funding streams are organised. Cluster 8 Researcher, US.

Interviewees from the UK also discussed the epistemic impact of research funding:

We just ignore the 90% of what causes inequalities and focus on the one bit where we can get money and we can have control. I can see how that actually impacts on me […] even though you know, fundamentally, it will only make a tiny bit of difference.

Cluster 4 Researcher, UK.

Partly as a reaction to the dominance of medical and disease-specific models in the US (Honjo, 2004), some US-based epidemiologists have advanced Social Epidemiology: “the study of how the social world influences–and in many cases defines–the fundamental determinants of health” (Berkman et al., 2014). These researchers, located in Cluster 3, helped cement social epidemiology as a legitimate sub-disciplinary specialisation (Galea and Link, 2013). Popular textbooks and key theoretical contributions to the field have been authored by members of Cluster 3, and the journal *Social Science and Medicine* (where many such contributions are published), is edited by members of Cluster 3. As these social epidemiologists are advancing a view of health as socially (rather than biomedically) situated, it is unsurprising that these scholars are located in the region of Cluster 3 closest to the social-science dominated network clusters.

## 4 Concluding discussion

Our bibliometric analysis confirms and for the first time visualizes researchers’ accounts of health equity research as a field comprised of clusters which are generally not well-connected. Our researcher profile and interview data suggest that, although disciplinary training played a role in the emergence of these clusters, so too have historical, geographic, institutional and financial (research funding) forces. Interpreting these results through the lens of Science and Technology Studies theorists Sheila Jasanoff and Thomas Kuhn helps to contextualise results within existing theoretical pictures of scientific progress.

### 4.1 A cartographic model of multi-discipline research

Jasanoff (2012) described two cartographic models of multidisciplinary research. The first, as nations within a continent, uninterrupted territory divided among disciplinary ‘states’, without space in between. The second, as an archipelago: wherein “the disciplines are oddly and idiosyncratically bounded formations, haphazardly scattered across a sea of ignorance, with unexplored waters in between.” (p192). In the continental model, disciplinary boundaries are broken by the development of *interstate highways*, in the archipelago model, by *voyages into the unknown*. Health equity research, as visualised in Fig. 1, Fig. 2, appears to display characteristics of both models, as it presents a central land-mass without gaps (Clusters 1, 4, 7 and 3), as well as peninsulas (Clusters 6 and 2), and islands (Clusters 5 and 8).

Social epidemiology might be described as a ‘highway’ between the biomedically-dominated, right-hand-side of the network and the social-science-rich left hand side. The appearance of social epidemiology as a distinct paradigm within mainstream epidemiology appears to be holding the two research communities together in citation-space, as this interdisciplinary specialty supports the interweaving of diverse perspectives. However, the ‘bridge’ researchers in Cluster 3 do not have strong links to the critical, policy-focused strand of health inequalities research produced by authors in Cluster 4, pointing to an apparent gap between US-led research on social epidemiology and critical policy analysis.

Collaboration on large-scale projects, and shared historical framings of equity pave the intellectual carriageways linking clusters 1, 2, 4 and 7. A shared conceptualisation of health as socially-situated links Clusters 6, 4 and 1. Cluster 2 is a well-connected methodological hub, and its central position reflects the high value placed on quantitative methodology across the field.

The ‘unexplored waters’ of health equity scholarship appear to lie in the gulf between (mainly) European scholars of the relationship between health and social class and the (mainly) US scholars of the intersection between health and race/ethnicity. Despite strong links to the epidemiologically-driven Cluster 1, Cluster 3 is poorly connected to the social science-dominated clusters 6, 7 and 4. We have shown that linguistic differences (‘inequalities’ vs ‘disparities’) do not fully explain this lack of connectivity, and are rather themselves a reflection of distinct, mature research traditions, each with their own history, disciplinary character, and funding landscape.

### 4.2 The long shadow of exemplary past achievement and the centrality of research funding

Although our findings make clear that disciplinary training alone cannot explain the clusters comprising health equity research, disciplinary siloing is clear. Thomas Kuhn's presentation of science as proceeding within independent paradigmatic communities (1962) highlights the ways in which early scholarly achievement powerfully shapes the trajectory of enquiry within disciplines. Kuhn's sense of disciplinary paradigms as “exemplary past achievement” (postscript to Kuhn, 1970) seems relevant, as early framings of health equity in the 1980s have cast long temporal shadows across Fig. 1. These early framings also appear to have influenced the attraction and recruitment of disciplinary specialists to the field; in the UK, where health inequalities research was politically controversial, sociology and political science are well-represented, and now present established, independent traditions of health equity scholarship. In the US, where health disparities began with unexplained variation in clinical practice, the clinical disciplines such as medicine, nursing, clinical epidemiology and psychology are more dominant, and ‘cancer disparities’ has emerged as a free-standing research domain, partly in response to relevant independent funding streams. The Kuhnian view warns against the prospect that these differences might be bridged simply, via multi-disciplinary teams, given the challenges of working across disciplines with fundamentally different paradigmatic assumptions. Indeed, future research might explore how researchers' ontological or epistemological outlooks impact on collaboration within health equity research, and public health more broadly.

Cutting across these historical, geographical and disciplinary norms are challenges relating to research funding. Our analysis highlights the importance of funding in shaping research content, and illustrates how 21st Century work reflects path-dependencies established many decades prior, in response to data access challenges and/or the design of foundational studies. Reflecting previous findings (Garthwaite et al., 2016; Smith, 2010), interview data illustrate that health equity researchers consider what is likely to be funded when making decisions about the direction of their work, and this appears to pull researchers towards disease-specific questions. Overall, we suggest that the formation of insular scholarly sub-networks within health equity research may be a consequence of the ways in which broad, motivating questions are rendered both scientifically manageable, and fundable.

Returning to the critiques outlined in our Introduction, an optimistic account might suggest that, having provided a visual and descriptive map of the field it will be possible for researchers to enhance cross-cluster dialogue, by engaging with work in clusters with which they are unfamiliar. It might also be feasible for interested funders to further incentivise the development of such interdisciplinary links. However, our analysis highlights the ways in which the complex nexus of factors underpinning the field's structure may also condition the field's content. With so many intersecting drivers, overcoming fragmentation means more than doing different work, it means being open and supported (institutionally and financially) to do work differently. The combined influences of funding, data availability, disciplinary norms and preferences, and national and institutional research cultures may render this flexibility difficult to achieve, in practice.

Likewise, interpreting the stable terminological division between ‘health inequalities’ and ‘health disparities’ research with an awareness of the performative nature of language in academia suggests this divide may be hard to bridge (i.e. use of particular terms to signal familiarity with, and to position new work in relation to what has gone before, and/or to demonstrate authors' normative values). Finally, as one reviewer reflected, competition over research funding can lead to researchers trying to protect their ‘territory’, as one interviewee's reference to ‘interlopers’ implied (see also Gieryn, 1983). Relatedly, these distinct territories of expertise may be serving distinct ‘clients’. Yet, without greater cohesiveness, lessons from policy studies suggests efforts to effect policy change to reduce health differences are likely to struggle (Smith, 2013).

At the time of writing, the unequal burden of COVID-19 across social groups is intensifying scholarly and public interest in health equity. In response to the pandemic, the need for studies focused on racial inequity in health in the US and UK has been highlighted (Bhala et al., 2020), suggesting that COVID-19 may represent a force capable of more closely uniting ‘health inequalities’ and ‘health disparities’ scholarship. However, while this study supplies an atlas for scholars to move beyond their existing networks, step outside the template of past successes, forge new alliances and explore less well-trodden paths, we have also detailed several reasons why researchers may be reluctant or unable to take these steps. Future studies might aid such efforts further, by examining the factors underlying this field's fragmentation in detail, including resources, the role of ontology, epistemology, disciplinary training and normative-political values in shaping researchers' perceptions of what is possible, what is feasible, and what is necessary to reduce observed inequity in health.

## Contribution statement

TC conceived and designed the study, undertook all data collection and analysis, and produced the first draft of the article. KS provided advice on the study design, data collection and analysis and contributed to drafting the final manuscript, notably the abstract, introduction and concluding discussion.
